# Machine learning-based prediction of BV-relevant probiotic functional potential in vaginal-derived *Lactobacillus crispatus* strains

**DOI:** 10.1186/s12866-026-04873-4

**Published:** 2026-03-17

**Authors:** Keyu Quan, Weichi Liu, Hao Jin, Weiwei Hu, Qinggele Borjihan, Qiuhua Bao, Yongfu Chen

**Affiliations:** 1 Inner Mongolia Key Laboratory of Dairy Biotechnology and Engineering, Inner Mongolia Agricultural University, Hohhot, Inner Mongolia China; 2 Key Laboratory of Dairy Products Processing, Ministry of Agriculture and Rural Affairs, Inner Mongolia Agricultural University, Hohhot, Inner Mongolia China; 3 Key Laboratory of Dairy Biotechnology and Engineering, Ministry of Education, Inner Mongolia Agricultural University, Hohhot, Inner Mongolia China

**Keywords:** Vaginal microbiota, *Lactobacillus crispatus*, Probiotic strain screening, Machine learning, Functional prediction

## Abstract

The vaginal microbiota plays a pivotal role in maintaining female reproductive health and homeostasis. However, most currently available *Lactobacillus* isolates have been developed for the prevention and treatment of intestinal diseases, whereas strain-specific investigations targeting female reproductive health remain limited. Conventional cultivation and screening strategies are often associated with high costs and substantial labor requirements. Machine learning enables rapid analysis of large-scale omics data, facilitating efficient identification of candidate probiotics. A total of 639 *Lactobacillus crispatus* isolates were recovered from vaginal secretions of healthy women using three selective media. Based on phylogenetic tree analysis, 67 representative strains were selected for in vitro assays including growth capacity, acidification capacity (pH reduction), lactic acid production, hydrogen peroxide production, and antimicrobial activity. A weighted scoring system was subsequently established based on these results, leading to the identification of three *Lactobacillus crispatus* strains with optimal overall performance. Finally, by integrating k-mer-based genomic features of the strains, a multi-stage feature selection strategy was employed in combination with multiple machine learning algorithms to develop a predictive pipeline, VLCPredictor. This pipeline is capable of scoring the functional potential of vaginal-derived *Lactobacillus crispatus* strains. Our study provides an efficient framework for the development and application of *Lactobacillus crispatus* in female reproductive tract health. It may support significant implications for maintaining microbial equilibrium in the female reproductive system, as well as for the prevention and treatment of gynecological infectious diseases.

**Supplementary Information:**

The online version contains supplementary material available at 10.1186/s12866-026-04873-4.

## Introduction

Microbial communities have co-evolved with humans for hundreds of thousands of years. Since birth, microorganisms adhere to various parts of the human body [1], such as the gut, skin, vagina, oral mucosa, lungs, and mammary glands, and the collective of these microorganisms is referred to as the microbiome. It is closely associated with human health and maintains key physiological functions of the host [2]. Among them, the vaginal microbiota is crucial for women’s reproductive health, maintaining vaginal health and homeostasis through nutrient competition, regulation of signaling molecules, and the production of bacteriocins, organic acids, and H₂O₂ [3–8]. Unlike the diverse microbial communities at other body sites, the vaginal microbiota of healthy reproductive-age women is typically dominated by a single *Lactobacillus* species, most commonly *Lactobacillus crispatus* (*L. crispatus*), *Lactobacillus iners* (*L. iners*), *Lactobacillus gasseri* (*L. gasseri*), and *Lactobacillus jensenii* (*L. jensenii*) [9–11]. Studies have indicated that vaginal microbiota dominated by *L. crispatus* tends to be more stable and healthier, whereas microbiota dominated by diverse anaerobes is generally considered indicative of vaginal dysbiosis [12]. Epidemiological studies have shown that long-term vaginal dysbiosis can trigger various diseases [13], such as vaginitis (e.g., BV) [14], sexually transmitted infections (e.g., HIV) [15–17], and genital herpes [18], while also increasing the risk of adverse pregnancy outcomes, including preterm birth [6, 19, 20] and miscarriage [21]. Therefore, the balance of the vaginal microbiota is essential for maintaining the normal physiological environment and the health of the vaginal ecosystem [22]. Notably, a balanced vaginal microbiota does not imply a permanently static ecosystem. Instead, the vaginal microbiome exhibits pronounced temporal dynamics driven by host physiological changes (e.g., the menstrual cycle) and external perturbations. Hugerth et al. collected daily vaginal swabs from 49 healthy young women over an entire menstrual cycle and used shotgun metagenomic sequencing to characterize day-to-day fluctuations in community composition. Their results showed that some individuals maintained a consistently *Lactobacillus*-dominated and relatively stable microbiota, whereas others experienced transient fluctuations around menses and even recurrent switching between *Lactobacillus*-dominated and dysbiotic states. Based on these longitudinal profiles, the authors proposed a classification framework for vaginal community dynamics (VCD), highlighting that even within the same community state type (CST), women can differ substantially in stability and community composition [23].

The research of vaginal microbiota has shown that specific bacterial communities, species, and strains can influence reproductive health. Restoring a beneficial microbial balance may represent an important strategy for protecting women’s reproductive health [24]. Since vaginal dysbiosis is associated with a reduction in *Lactobacillus* abundance, supplementation with exogenous *Lactobacillus* has emerged as a reliable and safe therapeutic approach [25–28]. Currently, some probiotic strains specifically targeting women's health have been developed, such as *L. crispatus* CTV-05 (Lactin-V). A randomized, double-blind, placebo-controlled Phase 2b clinical trial found that among 228 patients with bacterial vaginosis (BV) who received Metronidazole Vaginal Gel treatment, the recurrence rate was effectively reduced after 11 weeks of intervention with this strain [29]. Notably, previous studies have revealed significant genomic differences between *L. crispatus* isolated from healthy women and women with vaginitis [30]. Strains derived from healthy women more frequently harbor fragmented glycosyltransferase genes, suggesting that they may possess distinct ecological adaptation mechanisms under dysbiotic conditions. Zhang et al. administered *Lactobacillus rhamnosus* GR-1 and *Lactobacillus reuteri* RC-14 for 30 days to women with BV who had received intravaginal metronidazole suppositories for 7 days, and found that probiotic adjuvant therapy did not improve the cure rate compared with metronidazole treatment alone [31]. Although *L. iners* is one of the common lactic acid bacteria (LAB) in the vaginal microecosystem, unlike conventional dominant species such as *L. crispatus*, it typically becomes predominant during dynamic fluctuations of the vaginal microbiota (e.g., menstrual cycle or antibiotic use). Therefore, it is considered a “bridge species” in the process of microbial dysbiosis [32]. Prior reports have suggested that vaginal microbiota dominated by *L. iners* is associated with various reproductive health problems, including BV [33, 34]. These findings indicate that not all *Lactobacillus* strains are eligible for exogenous supplements, as they exhibit substantial differences in phenotypes and clinical effects depending on the strain and source of isolation. To date, a growing number of *L. crispatus* strains have been evaluated for probiotic-related properties. Previously characterized *L. crispatus* strains and their reported probiotic-associated features are summarized in Table S1. Although the high abundance of *L. crispatus* in the vagina has been associated with reproductive tract health, leading to its widespread use as a probiotic, but comprehensive studies on the taxonomic diversity are still lacking. Probiogenomic analysis of 97 *L. crispatus* strains revealed extensive genetic lineages within the same species, with strains isolated from the same vaginal sample dispersed across different clades [35]. These strains exhibited marked differences in ecological distribution and physiological functions, and no single COG was shared by all strains. Therefore, functional potential cannot be determined solely at the species level. Secondly, strains exhibit pronounced functional differences due to variations in their encoded genes. Even *L. crispatus* strains derived from the same ecological niche show significant differences in their capacity for glycogen degradation, which are closely associated with the structural integrity of pullulanase and its mRNA expression levels [36]. These differences ultimately lead to substantial variation in colonization ability and ecological adaptability among strains. Therefore, selecting truly effective and safe probiotics from the vast pool of candidate strains with substantial functional phenotypic differences remains a major challenge. It is also a complex and lengthy process that requires comprehensive investigations and evaluations to explore their viability and functional properties. With the advancement of genome sequencing technologies, a vast amount of microbial genomic data has become available. However, the lack of cultivation-based approaches and limited phenotypic information have severely restricted the in-depth exploration of the potential scientific value of probiotics. The development of new strategies for rapid probiotic screening and the effective evaluation of strain-level functional phenotypes has become a major focus of microbiome research.

In recent years, artificial intelligence (AI) and machine learning approaches have achieved transformative advances in the field of microbiome research, providing new strategies for probiotic strain screening [37]. These algorithms offer robust adaptability and ultra-high precision in deep analysis, enabling the rapid identification of probiotics with potential application value from massive omics datasets within a short time, thereby rendering traditional screening and characterization methods obsolete. Zhang et al. used k-mer as a feature extraction scheme and built a free online bioinformatics platform called “iProbiotics” based on the SVM algorithm [38]. By uploading the whole-genome sequence, the probability of a strain being a probiotic can be quickly and accurately predicted, serving as an auxiliary tool for experimental verification of potential probiotic strains. The construction of the platform has greatly reduced the cost and time required by researchers. Subsequently, another researchers developed “metaProbiotics”, a tool based on natural language processing (NLP) algorithms that can directly characterize probiotics among microbial communities by genomic sequences [39]. It enables the rapid discovery of novel probiotics from both metagenome-assembled genomes (MAGs) and genomes of isolated strains, and facilitates systematic investigations of their roles in maintaining host health. The aforementioned machine learning approaches have made significant progress in screening potential probiotics from massive databases. However, due to the scarcity of data linking genomes with phenotypes, it remains challenging to effectively discriminate between different strains within the same species. For instance, we input the *L. crispatus* isolates from this study into the iProbiotics and found that the scores were all high and difficult to distinguish (average score of 97.58%, Table S6).

Here, based on the phenotypic characteristics of 67 *L. crispatus* isolated from the vaginal microbiota of healthy women, we proposed a weighted scoring system and identified three strains with optimal overall performance. By integrating the k-mer features of the strain genomes, we applied a multi-stage feature selection strategy combined with multiple machine learning algorithms to construct a predictive pipeline, designated VLCPredictor, which enables scoring of *L. crispatus* isolates from the vagina in terms of acid production, lactic acid production, hydrogen peroxide generation, growth performance, and antagonistic activity against pathogens. In conclusion, our study not only provides technical support for strain screening in the development of microbial resources with potential to improve vaginal health, but also offers valuable *L. crispatus* strain-level insights to advance microbiome-based interventions for women’s health.

## Materials and methods

### Participants

A total of 68 women aged 18–30 years were recruited in Hohhot, Inner Mongolia, between November 2023 and January 2024. Participants were excluded if they met any of the following criteria: (1) History of vaginitis or recurrent vaginitis within the past 5 years; (2) Presence of cervical-vaginal sexually transmitted infection (*Chlamydia trachomatis*, *Neisseria gonorrhoeae*, *Mycoplasma genitalium*, or* Mycoplasma vaginalis*); (3) Positive HPV test; (4) History of recurrent vulvovaginal candidiasis or urinary tract infections; (5) Antibiotic use within the past three months, systemic medications, or probiotics (oral or intravaginal); (6) Acute illness; fallopian tube obstruction; any acute inflammatory condition; history of cancer; history of anorectal or genital malformations; history of anorectal or genital HPV infection; history of anorectal or genital herpes; vulvar or vaginal diseases (acute or chronic); abnormal urinalysis or urinary tract infection; or seropositivity for HIV, hepatitis C, hepatitis B, herpes simplex virus, or syphilis; (7) Current pregnancy, lactation, or a history of pregnancy; use of emergency contraception within the past year; or use intrauterine contraceptive devices. (8) Known hereditary or metabolic disorders. Ultimately, 36 women were included for vaginal secretion collection, and their health status was confirmed through routine blood and urine tests, gynecological examination, routine vaginal discharge examination, HPV and ThinPrep cytologic test (TCT) screening, as well as serological tests for common infectious diseases.

### Vaginal secretion sample collection and isolation of *L. crispatus*

All enrolled healthy women were instructed to provide samples during the non-menstrual phase and were required to meet the following criteria: (1) No vaginal irrigation within 48 h; (2) No sexual activity within the past week. Sample collection was performed at the Department of Obstetrics and Gynecology, Inner Mongolia People’s Hospital. Vaginal secretions were obtained by a gynecologist using a vaginal swab rotated against the lateral wall of the upper one-third of the vagina. If the secretion volume was insufficient, samples were collected from the posterior fornix. Samples were immediately placed on ice and transported to the laboratory, where they were subjected to serial dilution under aseptic conditions in clean bench. Aliquots of 200 μL from the 10⁻^3^, 10⁻^4^, and 10⁻^5^ dilutions were spread onto MRS, Rogosa, and BG agar plates, respectively. The BG medium was prepared by mixing Bryant and Burkey medium with mGAM medium at a 7:3 (v/v) ratio, as described by Donia et al. [40]. Plates were incubated anaerobically at 37 °C in an atmosphere containing 10% CO₂, 10% H₂, and 80% N₂ with 64% relative humidity for 48 h. Colonies displaying distinct sizes, morphologies, colors, and opacities were selected from the three types of media and subsequently purified, subcultured, and enriched. After washing off the residual medium with PBS, the bacterial pellet was collected. One portion was resuspended in skim milk and stored at − 80 °C, while the other was used for whole-genome sequencing.

### Whole-genome sequencing

Total DNA was extracted from culture pellets collected by centrifugation (8,000 × g, 5 min, 4 °C) using a DNA purification kit (TIANGEN, Beijing, China) supplemented with the STE buffer system, following the manufacturer’s instructions. The quality of the extracted DNA was assessed by agarose gel electrophoresis, and its concentration was determined using a Qubit® 2.0 fluorometer (Thermo Fisher Scientific, Waltham, USA). DNA samples meeting the quality criteria (concentration > 1 μg) were used for sequencing library construction. Quality assessment of the constructed libraries was performed using the Agilent 5400 system (Agilent Technologies, Santa Clara, USA) and Qubit® 2.0 fluorometer, with an expected average insert size of ~ 350 bp. Finally, 150-bp paired-end sequencing was performed on the Illumina NovaSeq X platform (Illumina, USA).

### *L. crispatus* genome assembly, annotation, and phylogenetic analysis

Raw sequencing data were quality-controlled using the in-house script klab_metaqc (https://github.com/jinhao94/klab_metaqc/) to remove low-quality bases and adapter sequences, generating high-quality clean reads. Clean data were assembled using SPAdes v4.0.0 with the parameter *–isolate*, and scaffolds shorter than 500 bp were removed using the Python script *18_select_target_length_seq.py* [41]. Genome quality was evaluated using the “lineage workflow” CheckM v1.1.0, with the criteria of completeness > 90% and contamination < 5% [42]. Genomes of all isolates were compared with reference genomes from the National Center for Biotechnology Information (NCBI) and the Genome Taxonomy Database (GTDB) using FastANI v1.33, and all isolates were classified into species-level clusters with an average nucleotide identity (ANI) of ≥ 95% [43].

Probiotic probability scores were estimated using the iProbiotics online predictor (https://www.imhpc.com/iProbiotics/webserver/public/index.html). The genome was annotated by Prokka v1.14.6 [44]. Carbohydrate-Active Enzymes (CAZymes) were identified using the dbCAN3 database with HMMER v3.3.2 [45]. Antibiotic resistance genes were identified using abricate v1.0.33 (https://github.com/tseemann/abricate) against the comprehensive antibiotic resistance database (CARD) [46]. Antimicrobial peptide (AMP) biosynthetic gene clusters were identified and annotated in the genomes using antiSMASH v7.0 [47]. The annotation of pullulanase (PulA) was primarily performed through the following steps: (1) Six PulA protein sequences reported in the literature [48] were compared against PulA protein sequences downloaded from the UniProt database [49], and sequences with > 35% similarity were retained, excluding those derived from environmental samples, bovine, human, or non-bacterial sources; (2) The remaining genomic protein sequences were then dereplicated at 90% similarity and 70% coverage, clustered, and representative sequences from each cluster were extracted to construct a PulA gene database. (3) All isolate genomes were annotated by BLASTp against the PulA gene database using an E-value threshold of 1e-5, and sequences with > 30% identity (pident) were considered PulA candidates.

Protein-coding genes were predicted using Prodigal v2.6.3 [50]. Based on PhyloPhlAn 3.0, a phylogenetic tree was constructed, and its confidence was evaluated through phylogenetic reconstruction using the RAxML maximum-likelihood program with 1,000 bootstrap replicates [51, 52]. Phylogenetic tree visualization was performed using iTOL v7.2.1 (https://itol.embl.de/).

### In vitro phenotypic and antimicrobial characterization of *L. crispatus*

A total of 67 representative *L. crispatus* strains were selected for phenotypic characterization based on genome analysis and phylogenetic tree branching results.

In each experiment, the following bacterial growth protocol was applied: all strains were retrieved from the −80 °C freezer, thawed, streaked onto solid agar plates, and incubated at 37 °C for 24–48 h with plates inverted. Subsequently, the cultures were transferred twice into liquid medium (2% inoculum) to ensure strain viability prior to experimentation. All experiments were conducted in triplicate.

#### Bacterial solid culture medium

*L. crispatus* was maintained on MRS Agar; *Gardnerella vaginalis* ATCC 14018 (*G. vaginalis*), *Fannyhessea vaginae* ATCC BAA-55 (*F. vaginae*) and *Prevotella bivia* ATCC 29303 (*P. bivia*) were maintained on Columbia blood agar (CBA, purchased as prepared media from Solarbio) supplemented with 5% fresh defibrinated sheep blood (Solarbio, Beijing, China). *Staphylococcus epidermidis* ATCC 14990 (*S. epidermidis*) was maintained on Tryptic Soy Agar (TSA, purchased as prepared media from Solarbio).

#### Bacterial broth culture media

*L. crispatus* was maintained on MRS broth, *G. vaginalis* ATCC14018, *F. vaginae* ATCC BAA-55 and *P. bivia* ATCC 29303 were maintained on Tryptic Soy Broth (TSB, purchased as prepared media from Solarbio) supplemented with 5% fresh defibrinated sheep blood; *S. epidermidis* ATCC 14990 was maintained on TSB.

#### Bacterial culture condition

*L. crispatus*,* F. vaginae*, and *P. bivia* were cultured aerobically (10% CO₂, 10% H₂, 80% N₂; 64% relative humidity) at 37 °C for 24–48 h. All culture media were pre-equilibrated overnight in the anaerobic workstation to ensure complete deoxygenation prior to use.

*G. vaginalis* was cultured at 5% CO₂ at 37° C for 48 h. *S. epidermidis* was aerobic cultured at 37 ℃ for 24 h.

#### Preparation of *L. crispatus* CFS

Working cultures grown for 20 h were centrifuged at 13,000 rpm and 4 °C for 5 min. The supernatant was subsequently filtered through a sterile 0.22 µm membrane filter to remove any remaining viable bacterial cells.

#### Preparation of *L. crispatus* bacterial pellet

Working cultures grown for 20 h were centrifuged at 3500 rpm and 4 °C for 5 min, followed by three washes with PBS to remove residual culture medium.

#### Determination of growth capacity

An aliquot (200 μL) of freshly sub-cultured bacterial suspension and sterile medium (blank control) were aspirated and transferred into a bioscreen C plate. OD600 was measured every hour for 24 h under aerobic conditions. The growth curve was drawn and the maximum specific growth rate (μmax) was calculated.

#### Determination of maximum acid production

The pH of *L. crispatus* CFS was measured using a pH meter.

#### Determination of hydrogen peroxide (H_2_O_2_)

The concentration of H_2_O_2_ in the cell-free supernatant (CFS) of *L. crispatus* was quantified by the Tetramethylbenzidine (TMB) colorimetric assay.

To construct the H_2_O_2_ standard curve, working solutions of 0, 20, 40, 60, 80 and 100 μmol L⁻^1^ H_2_O_2_ were prepared in 100 mmol L⁻^1^ PIPES buffer (pH 7.0) from a 30% (w/w) H_2_O_2_ stock solution (equivalent to 9.128 mol L⁻^1^). For each standard, 100 μL of the H_2_O_2_ working solution was mixed with 100 μL of 20 mM TMB solution, followed by the addition of 2 μL of horseradish peroxidase (HRP, 1 mg/mL). The mixture was incubated at 25 °C in the dark for 10 min, followed by OD600 measurement to generate a standard curve. Following the aforementioned procedure, the cell-free supernatant (CFS) of *L. crispatus* was assayed, and the concentration of H_2_O_2_ was calculated using the standard curve. All solutions, including PIPES buffer, TMB solution, and HRP, were prepared fresh immediately prior to use.

The hydrogen peroxide (H_2_O_2_) content in *L. crispatus* bacterial pellet was quantified using a titanium salt colorimetric method, in accordance with the manufacturer's instructions provided with the commercial assay kit (Ruixinbio, Fujian, China). The results are expressed as μmol H_2_O_2_ per gram of wet cell weight (μmol/g).

#### Determination of lactic acid

High-performance liquid chromatography (HPLC) was performed to quantify the concentration of lactic acid in the *L. crispatus* CFS.

For sample pretreatment, 40 μL of appropriately diluted CFS from *L. crispatus* was mixed with 20 μL each of two derivatization reagents: 200 mM 3-NPH solution and 120 mM EDC. The mixture was vortexed thoroughly and incubated at 40 °C for 30 min. The reaction was quenched by adding 1.92 mL of 10% (v/v) acetonitrile in water, followed by centrifugation at 4 °C and 12,000 × g for 10 min. Subsequently, 500 μL of the supernatant was collected, filtered through a 0.22 μm sterile membrane, and transferred into an HPLC vial for subsequent analysis.

The chromatographic column temperature was 40 ℃. Mobile phase A is ultrapure water containing 0.1% formic acid, and mobile phase B is methanol containing 0.1% formic acid. The gradient was as follows: 0.0–1.0 min, 5.0% B; 1.0–6.5 min, 5.0–100.0% B; 6.5–9.5 min, 100.0–100.0% B; 9.5–11.0 min, 100.0%−5.0% B; 11.0–12.0 min, 5.0% B; flow rate: 0.4 mL/min; injection volume: 1 µL.

Mass spectrometric detection was performed using a SCIEX QTRAP® 6500 + triple quadrupole mass spectrometer operated in multiple reaction monitoring (MRM) mode. The key parameters were configured as follows: curtain gas pressure was set to 25 psi, ion source auxiliary gas 1 and gas 2 were both maintained at 50 psi, and the source temperature was held at 550 °C. The electrospray ionization (ESI) source voltage was set to + 5500 V in positive ion mode and − 4500 V in negative ion mode. High-purity nitrogen was used as the collision gas.

A series of standard solutions at varying concentrations were prepared, and the corresponding chromatographic peak areas were measured for each concentration. A standard curve was constructed by plotting the concentration of the standard solutions on the x-axis against the peak area on the y-axis. The integrated peak area obtained from the sample was then substituted into the linear regression equation of the standard curve to calculate the corresponding concentration. The value was further converted to the actual content of the compound in the sample by incorporating parameters such as sample volume and dilution factor.

Raw data were acquired using Analyst® 1.7.1 software, while the construction of calibration curves and quantitative analysis of samples were performed using SCIEX OS-Q™ software.

#### Antimicrobial activity

To evaluate the inhibitory effect of *L. crispatus* against common pathogenic bacteria associated with vaginal dysbiosis, four type strains known to frequently disrupt vaginal microbiota homeostasis were selected:* G. vaginalis* ATCC 14018, *S. epidermidis* ATCC 14990, *F. vaginae* ATCC BAA-55, and *P. bivia* ATCC 29303. The inhibition assays were performed in accordance with the EUCAST standard for antimicrobial susceptibility testing, utilizing the agar diffusion method. Briefly, the bacterial suspension was adjusted to an optical density equivalent to 0.5 McFarland standard, ensuring the concentration of approximately 1–2 × 10⁸ CFU/mL. Sterile cotton swabs were immersed in the pathogenic bacterial suspension, then rotated and pressed against the inner wall of the tube to remove excess liquid. The plate was evenly spread along the spread evenly in three directions to form a uniform lawn (rotated 60° each time). After allowing the bacterial suspension to dry on the surface, wells were punched in the agar. Subsequently, the CFS and bacterial pellet of *L. crispatus* were added to separate wells. All procedures were completed within 15 min.

### Composite functional score and weighting system

To generate class labels for model training, a composite function score was calculated by integrating multiple in vitro phenotypes (Table S3). Before starting, all phenotypic values were normalized. The composite score was computed as a weighted sum of the normalized phenotypes using fixed weights (sum = 1.0): growth capacity (0.1), acidification (culture pH; 0.2), total lactic acid production (0.2), H₂O₂ production (0.2), and antagonistic activity against BV-associated pathogens (0.3).

### Model construction

In our study, a machine learning framework named VLCPredictor, which integrates multi-stage feature extraction, stability-enhanced feature selection, and multi-model comparative prediction, was proposed for sequence classification tasks. The overall workflow consists of three main steps: feature representation, feature selection, and model prediction.

#### Feature representation

In the feature representation stage (A: Feature Representation, Fig. 2), multi-order k-mer features were extracted from the original nucleic acid sequences. A k-mer refers to a substring of length k, whose frequency is counted using a sliding window approach. Assuming the input sequence is a string S = s₁s₂s₃⋯s_L with length L, a total of L—k + 1 fragments of length k can be extracted. In this study, k was set to range from 5 to 9 (k ∈ {5, 6, 7, 8, 9}). The frequency vector for each k-order was calculated separately, and the features of all orders were concatenated to form the final high-dimensional feature vector:

1 $$x= \left[{x}^{\left(5\right)}\Vert {x}^{\left(6\right)}\Vert {x}^{\left(7\right)}\Vert {x}^{\left(8\right)}\Vert {x}^{\left(9\right)}\right]$$

where ${x}^{(k)}$ denotes the frequency distribution vector of k-mers, and $\|$ represents the vector concatenation operation. The generated feature matrix has a dimension of 349,184, which exhibits extremely high sparsity and dimensional redundancy, thus requiring further screening.

To mitigate the impact of imbalanced feature distribution, all features were standardized using the z-score method:

2 $${x}_{ij}^{\prime} = \frac{{x}_{ij}-{\mu }_{j}}{{\sigma }_{j}}$$

where ${x}_{ij}$ is the original value of the i-th sample on the j-th feature, and ${\mu }_{j}$ and ${\sigma }_{j}$ are the mean and standard deviation of the j-th feature, respectively.

#### Feature selection

To reduce the computational burden and overfitting risk caused by the high-dimensional feature space, a multi-stage, stability-enhanced, and redundancy-controlled feature selection method was designed and implemented in this study (B: Feature Selection, Fig. 2). This method integrates strategies such as statistical filtering, score fusion, and correlation-based clustering pruning to ensure that the finally selected features demonstrate excellent performance in terms of information content, stability, and non-redundancy.

1. Initial screening: low-variance filtering and univariate scoring

In the initial stage, the VarianceThreshold method was first used to remove feature dimensions with little variation in the training set. The discrimination criterion is as follows:

3 $$\mathrm{Var}\left({X}_{j}\right)=\frac{1}{n}{\sum }_{i=1}^{n}{\left({x}_{ij}-{\overline{x}}_{j}\right)}^{2}<\upvarepsilon$$

where ${X}_{j}$ represents the j-th feature vector, ${x}_{ij}$ is the value of sample i on feature $j,{\overline{x} }_{j}$ is the sample mean of feature j, n is the total number of samples, and $\varepsilon ={10}^{-5}$ is an empirically set threshold.

Subsequently, Analysis of Variance (ANOVA) was employed for feature ranking. This method evaluates the statistical association between each feature and the label by calculating the ratio of between-class variance to within-class variance (i.e., F-value):

4 $${F}_{j}=\frac{{\mathrm{MSB}}_{j}}{{\mathrm{MSW}}_{j}}$$

5 $${\mathrm{MSB}}_{\mathrm{j}}=\frac{1}{\mathrm{C}-1}{\sum }_{\mathrm{C}=1}^{\mathrm{C}}{{\mathrm{n}}_{\mathrm{C}}\left({\overline{\mathrm{x}} }_{\mathrm{i},\mathrm{c}}-{\overline{\mathrm{x}} }_{\mathrm{j}}\right)}^{2}$$

6 $${\mathrm{MSW}}_{j}=\frac{1}{n-C}{\sum }_{C=1}^{C}{\sum }_{i\in c}{\left({x}_{ij}-{\overline{x} }_{j,c}\right)}^{2}$$

where ${\mathrm{MSB}}_{j}$ is the between-class mean square, ${\mathrm{MSW}}_{j}$ is the within-class mean square, C is the number of classes, ${n}_{c}$ is the number of samples in the c-th class, and ${\overline{x} }_{j,c}$ is the mean of feature j in the c-th class. By selecting the top K features with the largest F-values (up to 10,000), feature dimensions that may be significantly correlated with the label distribution were retained.

2) Stability-enhanced multi-criteria fusion scoring

To further improve the robustness and discriminative power of feature selection, a weighted scoring mechanism that integrates multiple evaluation metrics was introduced in this study, combined with resampling techniques to enhance robustness against perturbations in the training data. In each iteration, four types of feature importance metrics were calculated separately: F-value (for evaluating linear separability); Mutual Information (MI, for measuring non-linear correlations); Chi-squared Test (for testing the independence of categorical variables); Random Forest Importance (based on the feature splitting contribution of tree-structured models). Since these scores vary in their numerical ranges, they were uniformly normalized using the min–max normalization method:

7 $${S}_{j}^{\left({k}\right)}=\frac{{S}_{j}^{\left({k}\right)}-\mathrm{min}\left({S}^{\left({k}\right)}\right)}{\mathrm{max}\left({S}^{\left({k}\right)}\right)-\mathrm{min}\left({S}^{\left(t{k}\right)}\right)+\upepsilon }$$

where ${S}_{j}^{(k)}$ is the normalized result of feature j in the k-th scoring metric, and $\epsilon$ is a small positive number used to avoid division by zero.

The fused score was defined as:

8 $${S}_{j}^{\mathrm{fused}}= \alpha \bullet {F}_{j}^{\prime} + \beta \bullet\;\mathrm{MI}_{j}^{\prime} + \gamma \bullet {X}_{j}^{{\prime}2}+\delta \bullet\;\mathrm{RF}_{j}^{\prime}$$

Where ${F}_{j}{\prime}$, ${\mathrm{MI}}_{j}{\prime}$, ${X}_{j}^{{\prime}2}$, and ${\mathrm{RF}}_{j}{\prime}$ represent the normalized values of the above four types of scores, respectively. The weight coefficients were set as $\alpha =0.3$, $\beta =0.3$, $\gamma =0.2$, and $\delta =0.2$ to emphasize the dominance of statistical and information-theoretic indicators.

This process was conducted for N = 5 rounds of bootstrap resampling. In each round, 80% of the training data was sampled, and the above fused scores were calculated. The final feature score was the average across all rounds:

9 $${\overline{S} }_{j}=\frac{1}{N}{\sum }_{r=1}^{N}{S}_{j}^{\mathrm{fused},\left({r}\right)}$$

Features were ranked according to their scores, and the top R% (with R = 80% by default) of the features were selected for further processing in the next stage.

3) Redundant feature elimination based on correlation

Due to the severe collinearity often present in high-dimensional features, a hierarchical clustering method based on absolute correlation coefficients was adopted in this study to further eliminate redundant features. First, the Pearson correlation coefficient matrix between each pair of selected features was calculated:

10 $${\rho }_{ij} = \left|\frac{\mathrm{Cov}\left({X}_{j},{X}_{j}\right)}{{\sigma }_{i}{\sigma }_{j}}\right|$$

Then, a symmetric distance matrix was constructed:

11 $${D}_{ij}=1-{\rho }_{ij}$$

A smaller value of this distance metric indicates a higher level of redundancy between features. The average linkage method was applied to the distance matrix for hierarchical clustering, and clustering was cut off by setting a distance threshold.

For each feature cluster, the feature with the highest fused score was retained as the representative feature. Finally, a subset of 534 features was obtained from the original 349,184 features, which balances information content, stability, and redundancy reduction.

#### Model prediction

In the model prediction stage, the entire dataset was first divided into a training set and a test set in a ratio of 60%:40% using stratified sampling to maintain a consistent class distribution. The training set was used for model construction and validation optimization, while the test set was used to evaluate the generalization performance of the final model. To comprehensively compare the performance of different classification strategies, eight common classifiers were selected, including Logistic Regression (LR), Support Vector Machine (SVM), Random Forest (RF), Naive Bayes (NB), K-Nearest Neighbors (KNN), Decision Tree (DT), XGBoost (XGB), and LightGBM (LGB). These models cover typical algorithms such as linear discrimination, kernel methods, tree models, probabilistic models, and ensemble learning, which can effectively handle diverse data features and distributions.

The model training adopted the Repeated Holdout Validation strategy. The training set was randomly divided into validation sets for 10 rounds, each model was trained and evaluated multiple times, and the average performance metrics were calculated. The Area Under the ROC Curve (AUC) was used as the evaluation criterion to measure the model's ability to distinguish between positive and negative samples. AUC reflects the overall performance of the model under different decision thresholds, with a value closer to 1 indicating better classification performance. Finally, the models were ranked based on their average AUC on the training set, and the optimal model was selected for testing on the independent test set. The entire workflow ensures the stability of model selection, the objectivity of evaluation, and the reproducibility of the final results.

## Results

### Vaginal *Lactobacillus* isolate culture group

**Fig. 1:**
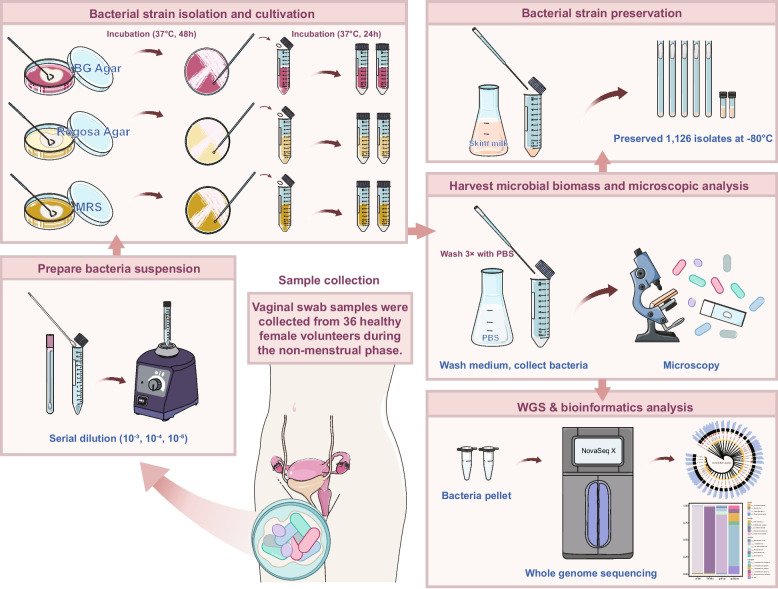
Workflow for isolation, preservation, and genomic characterization of vaginal bacterial isolates. Vaginal swabs were collected from 36 healthy women during the non-menstrual phase. Bacterial isolates were recovered on selective agar media, purified, and examined microscopically. Each isolate was divided into two aliquots: one cryopreserved in skim milk at − 80 °C for long-term storage, and the other subjected to whole-genome sequencing (WGS) and subsequent bioinformatics analysis

The study evaluated the eligibility of 68 candidate volunteers. After excluding those who did not meet the inclusion criteria, refused to participate, or were unable to participate for other reasons, vaginal secretion samples were collected from the remaining 36 healthy women. To accumulate *L. crispatus* isolates, samples were serially diluted in PBS and evenly spread onto three specific solid media (MRS, Rogosa Agar and BG Agar). These plates were incubated anaerobically for 48 h, after which strains exhibiting different morphologies were picked for isolation and purification. Whole-genome sequencing was performed on all strains, resulting in the isolation and preservation of 1,126 bacterial strains (Fig. 1).

**Fig. 2:**
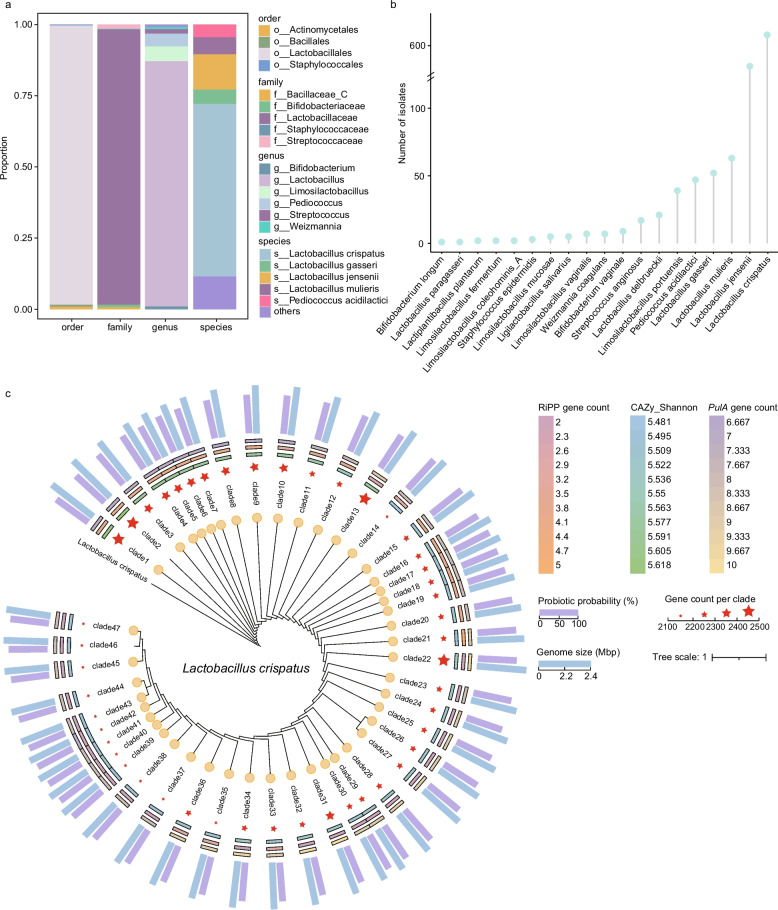
Genomic diversity and phylogenetic structure of vaginal bacterial isolates. **a **Taxonomic composition of 1,053 genomes based on 95% average nucleotide identity (ANI), clustered into 20 species-level groups spanning 4 orders, 5 families, 6 genera, and 19 species. **b** Species-level distribution of isolates, showing that the majority (78.06%) were *Lactobacillus* species, including *L. crispatus* (639 isolates), *L. jensenii* (131 isolates), and *L. gasseri* (52 isolates). **c** Phylogenomic analysis of 639 *L. crispatus* genomes revealed 47 distinct clades. In the circular phylogeny, blue bars indicate genome size, and purple bars represent predicted probiotic potential as estimated by the iProbiotics platform. The heatmap rings, from outer to inner, represent the number of encoded amylases, the number of antimicrobial peptides, and the Shannon diversity index of carbohydrate-active enzymes, respectively. Red stars indicate gene count per clade

After de novo assembly of sequencing reads, 1053 genomes were obtained. Through genome quality assessment, the average completeness was 99.91% ± 0.3, and the contamination was 0.33% ± 0.36. Subsequently, using a 95% average nucleotide identity (ANI) threshold, the 1,053 genomes were delineated into 20 species-level clusters, which were taxonomically assigned to 4 orders, 5 families, 6 genera, and 19 species (Fig. 2a). The majority of the isolates (78.06%) were assigned to the common LAB in the vagina, including *L. crispatus* (639 isolates), *L. jensenii* (131 isolates), and *L. gasseri* (52 isolates) (Fig. 2b, Table S2). Previous studies have shown that *L. crispatus* plays an indispensable role in maintaining the health of the female reproductive tract, and its abundance is regarded as an important biomarker of vaginal health. Therefore, subsequent analyses primarily focused on the 639 *L. crispatus* isolates obtained from vaginal samples.

### Phylogenomics and representative *L. crispatus* selection

A phylogenetic analysis was performed on 639 *L. crispatus* (Fig. 2c), and a total of 47 clades were obtained, among which the largest branch was clade_12 (containing 163 strains, accounting for 25.6%). The average genome size across the 47 clades was 2.16 Mbp (range: 2.03–2.40 Mbp). The mean GC content was 36.99%, and an average of 2,252 (2106–2494) genes were annotated per clade. The *L. crispatus* pan-genome consisted of 4,482 different gene families. The core genome consisted of 1579 genes (35.23% of total) and 601 accessory genes per strain (20.7% of total). Among the 47 clades, 12 possessed unique genes (25.5%), with significant variations in distribution across branches. Based on genomic features previously associated with functional potential, we conducted a stepwise screening based on the presence or absence of antibiotic resistance genes, the type and copy number of encoded pullulanase, the number of antimicrobial peptide gene clusters, the probiotic probability predicted by the iProbiotics, the genome score (Completeness − 5 × Contamination + log₁₀ N50), as well as the repertoire of encoded carbohydrates. One representative strain was selected from each clade, yielding 47 strains that collectively captured 47.15% of the pan-genome gene families. However, these strains were derived from only 13 individuals, representing 50% of the cohort. To ensure host representativeness, 20 *L. crispatus* strains were further selected from different individuals across the branches according to the above criteria, collectively encompassing 46.8% of the pan-genome.

### Characterization of physiological, biochemical, and antimicrobial properties across *L. crispatus* lineages

**Fig. 3:**
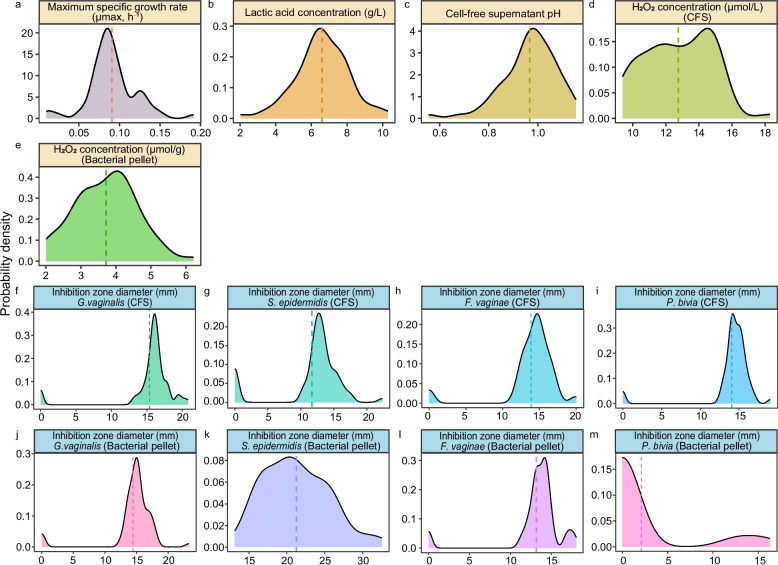
Density plots illustrate the distribution of five key phenotypic traits across 67 isolates. The x-axis represents the measured value for each trait: **a** maximum specific growth rate (μmax, h⁻^1^), **b** lactic acid concentration (g/L), **c** cell-free supernatant (CFS) pH, and **d**, **e** H₂O₂ concentration (μmol/L for CFS; μmol/g for bacterial pellets), and inhibition zone diameter (mm) against four pathogens (**f**-**m**): *G. vaginalis*, *S. epidermidis*,* F. vaginae*, and *P. bivia* assessed using both CFS and bacterial pellets. The y-axis represents the probability density, reflecting the relative frequency of isolates at each value. Dashed vertical lines indicate the median value for each trait

The overall genomic structure within the same species is highly conserved. Relying solely on genomic information makes it difficult to effectively distinguish the differences in functional potential between the strains. Consequently, we determined the five phenotypes including growth ability, acid production, lactic acid production, H_2_O_2_ production (cell-free supernatant, CFS and bacterial pellet) and antagonistic activity against pathogenic bacteria (CFS and bacterial pellet) based on the inherent core beneficial characteristics of *L. crispatus* in the vagina. Turns out the variation range of these strains in different phenotypes was large, details are as follows (Fig. 3, Table S3).

### *L. crispatus* growth kinetics

The growth performance reflects adaptability and competitive capacity, serving as a key determinant of whether it can colonize the vagina and exert sustained effects. Moreover, it is also one of the primary criteria for the selection of probiotic candidates in formulation development and practical applications. The growth dynamics of 67 *L. crispatus* strains were observed in MRS culture medium with pH 6.0 under microaerobic conditions at 37 °C. Most strains entered the exponential phase within 2–4 h, which persisted until 8–12 h, after which growth gradually plateaued around 20 h. Furthermore, growth curves were determined for each strain, and the maximum specific growth rate (μ_max_) was calculated by fitting the exponential phase of OD_600_. The μ_max_ of the isolates ranged from 0.01–0.19 h⁻^1^, indicating that most strains exhibited favorable growth capacity and adaptability (Fig. 3a).

### *L. crispatus* acid production capacity

The vaginal environment of healthy women typically maintains a slightly acidic environment, with pH 3.8–4.5. Following anaerobic incubation of all strains for 20 h, the endpoint pH value of all strains CFS was assessed using a calibrated pH meter. The results showed that the pH range of strain CFS was 3.8–4.45 (4.03 ± 0.11), indicating that all strains exhibited robust acidification capacity (Fig. 3c).

### H_2_O_2_ production

Prior studies have shown that *L. crispatus* may inhibit the growth of anaerobic bacteria through H_2_O_2_ production and is therefore associated with vagina health. We used a colorimetric method to determine the H_2_O_2_ content of 67 *L. crispatus* strain culture supernatants in CFS and after bacterial fragmentation, respectively. The CFS of all strains cultures contained 9.39–18.3 μmol/L H_2_O_2_, with the highest being iLABdb.g73758 (18.3 μmol/L), followed by iLABdb.g73559 (15.62 μmol/L) and iLABdb.g73280 (15.14 μmol/L) (Fig. 3d). The bacterial pellet contained relatively small amounts of H_2_O_2_ content ranging from 2.01–6.21 μmol/g. However, the CFS did not show any linear relationship with the H_2_O_2_ content in the bacterial pellet, which was highest in iLABdb.g73868 (6.22 μmol/g), followed by iLABdb.g73486 (5.31 μmol/g) and iLABdb.g73192 (5.31 μmol/g) (Fig. 3e).

### Lactic acid production

Lactic acid is considered a major metabolic product of *L. crispatus*, exerting significant antimicrobial activity. Therefore, the lactic acid production of 67 *L. crispatus* strains was evaluated. All strains were grown in MRS broth for 24 h and then the lactate content was assessed by high performance liquid chromatography (HPLC). Results revealed that lactic acid production averaged 6.6 g/L for all strains (range 2.02–10.29 g/L). The strain with highest lactic acid content is iLABdb.g73605 (10.29 ± 0.61 g/L), followed by iLABdb.g73395 (9.85 ± 0.69 g/L) and iLABdb.g73151 (9.39 ± 0.35 g/L) (Fig. 3b).

### Ability to inhibit pathogenic bacteria

Finally, the inhibitory effects of 67 *L. crispatus* strains against vaginal pathogens were evaluated. This phenotype directly reflects the ability of the strains to suppress pathogenic bacteria, which is a critical determinant of their potential in the treatment of Vaginitis. Representative pathogenic strains were selected according to the major types of vaginitis, including BV and aerobic vaginitis, namely *G. vaginalis* ATCC 14018, *F. vaginae* ATCC BAA-55, *P. bivia* ATCC 29303, and *S. epidermidis* ATCC 14990. In the light of the EUCAST antimicrobial susceptibility testing guidelines, the agar diffusion method was employed to evaluate the antagonistic activity of both the CFS and bacterial pellets of *L. crispatus* against the four pathogenic strains. The results showed that the majority of *L. crispatus* strains exhibited inhibitory effects against *G. vaginalis* (CFS: 15.35 ± 4.12 mm; bacterial pellets: 14.41 ± 3.96 mm), *F. vaginae* (CFS: 13.86 ± 3.85 mm; bacterial pellets: 13.15 ± 3.61 mm), and *S. epidermidis* (CFS: 11.66 ± 4.96 mm; bacterial pellets: 21.27 ± 4.25 mm) (Fig. 3j –m). In contrast, although the CFS of most strains displayed inhibitory activity against *P. bivia* (14.04 ± 3.23 mm), only a minority of strains demonstrated detectable inhibition through their bacterial pellets (Fig. 3f–i).

### Genomic and key phenotypic features-based VLCPredictor enables accurate prediction of superior strains

**Fig. 4:**
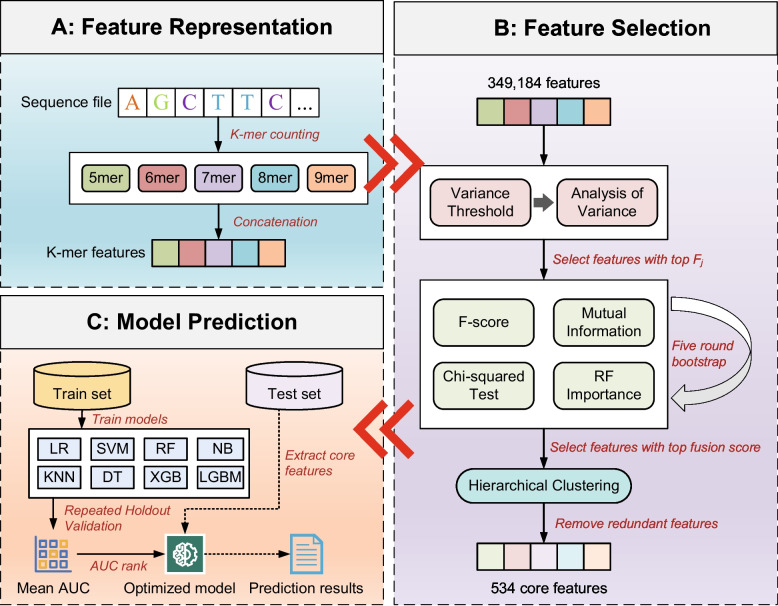
Architecture of the VLCPredictor machine learning framework. **a** Feature representation: Whole-genome sequences were decomposed into 5–9-mers, and their frequencies were concatenated to form a high-dimensional feature vector (349,184 dimensions). **b** Feature selection: A multi-stage pipeline reduced dimensionality: (i) low-variance and analysis of variance-based filtering, (ii) fusion of four importance metrics (*F*-score, mutual information, chi-squared, random forest (RF)), and (iii) hierarchical clustering to remove correlated features, yielding 534 non-redundant, stability-enhanced features. **c** Model prediction: Eight machine learning algorithms, including logistic regression (LR), support vector machine (SVM), RF, naïve Bayes (NB), K-nearest neighbors (KNN), decision tree (DT), extreme gradient boosting (XGB), and light gradient boosting machine (LGBM), were trained on the refined feature set; model performance was evaluated by repeated holdout validation, and the optimized-performing model (RF) was selected based on mean area under the receiver operating characteristic curve (AUC) for final deployment

**Fig. 5:**
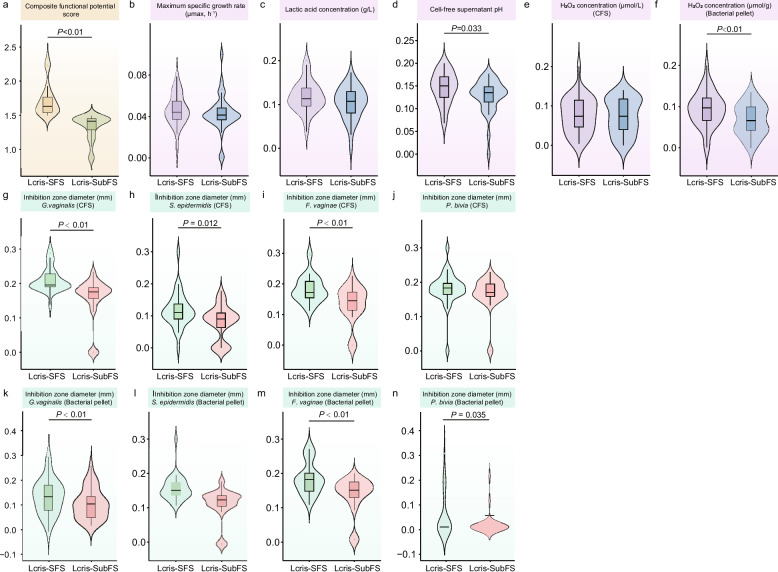
Comparison of functional performance between Lcris-SFS and Lcris-SubFS. Violin plots display the distribution of the composite probiotic function score and normalized scores of individual functional traits across the two groups. Each violin represents the kernel density estimate of the data, with wider sections indicating higher density of observations. Embedded within each violin is a boxplot: the horizontal line denotes the median, the box spans the interquartile range (IQR; 25th to 75th percentile), and the whiskers extend to the most extreme data points within 1.5 × IQR of the box edges. Two group comparisons are shown for the (**a**) composite functional potential score and normalized individual traits, including (**b**) maximum specific growth rate (μmax, h⁻^1^), (**c**) lactic acid concentration (g/L), (**d**) cell-free supernatant (CFS) pH, and (**e**, **f**) H₂O₂ concentration (μmol/L for CFS; μmol/g for bacterial pellets), and inhibition zone diameter (mm) against four pathogens (**g**-**n**): *G. vaginalis*, *S. epidermidis*,* F. vaginae*, and *P. bivia* assessed using both CFS and bacterial pellets. *P* values are indicated above each panel and were calculated using two-sided Mann–Whitney U tests

To systematically screen *L. crispatus* strains with high functional potential, we developed a machine learning-based predictive pipeline, designated VLCPredictor, based on in vitro functional trait assessments (Fig. 4). VLCPredictor links the genomic k-mer features of the strains with composite labels derived from multiple phenotypic traits. Due to the limited sample size, accurate prediction using continuous variables remains challenging. Consequently, we first established a weighted scoring system (Table S3) to screen for potential high-quality strains, based on a comprehensive review of the literature and the practical efficacy of LAB in vaginal applications. Before applying feature selection algorithms, all data were normalized. The weights assigned to the phenotypic indicators were constrained to values between 0 and 1 and summed to 1, with larger weights reflecting higher importance and priority of the corresponding strain traits. Since the *L. crispatus* used in this study were isolated from the vagina of healthy women, it was assumed that they would adapt well to the vaginal environment, and the growth differences would have minimal impact on the subsequent screening of superior strains. Therefore, the weight assigned to growth capacity was set at 0.1. The production of acid, lactic acid, and H_2_O_2_ are common inhibitory mechanisms by which *L. crispatus* suppresses the growth of pathogenic microorganisms. In vaginal environment, these factors can also exert direct or indirect effects on host tissues by modulating immune responses and gene expression. Accordingly, each of these traits was assigned a weight of 0.2. Antagonistic activity represents the most effective and direct indicator of the inhibitory potential of *L. crispatus* against pathogens, and was therefore assigned the highest weight of 0.3. The overall score of each strain was calculated by weighted summation. As the score distribution was continuous and lacked a clear biological threshold, the median value was adopted as the cutoff. Strains ranking in the top 50% were defined as “Lcris-SFS” (Superior Functional *L. crispatus*), while those in the bottom 50% were designated as “Lcris-SubFS” (Suboptimal Functional *L. crispatus*). Lcris-SFS demonstrated significantly better overall performance across the five evaluated phenotypes compared with the Lcris-SubFS (Fig. 5a), particularly in acid production (Fig. 5d), hydrogen peroxide production (cells, Fig. 5e), and inhibition of *Gardnerella vaginalis* (CFS and bacterial pellet, Fig. 5g, k), *Staphylococcus epidermidis* (bacterial pellet, Fig. 5h), *Fannyhessea vaginae* (CFS and bacterial pellet, Fig. 5i, m), and *Prevotella bivia* (bacterial pellet, Fig. 5n) (*P* < 0.05, Mann–Whitney U test). Moreover, based on this weighted scoring system, three *L. crispatus* strains with the highest functional potential (iLABdb.g73486, iLABdb.g73294, and iLABdb.g73153) were identified. These strains are currently being further evaluated in animal models and clinical studies to validate their in vivo efficacy and underlying mechanisms.

In the first stage, the VLCPredictor framework was developed and internally evaluated using a labeled dataset comprising 67 *L. crispatus*. Within this framework, genomic sequences were first represented using k mer based features, followed by a multi round feature selection procedure to identify informative genomic signals. Based on the selected features, a downstream prediction module was implemented in which eight commonly used machine learning algorithms were applied in parallel, including logistic regression (LR), support vector machine (SVM), random forest (RF), naïve Bayes (NB), k-nearest neighbor (KNN), decision tree (DT), XGBoost (XGB), and LightGBM (LGBM). For internal evaluation, the labeled dataset was randomly divided into training and testing subsets at a ratio of 6:4, and the entire process was repeated 10 times using different random seeds to ensure robust and unbiased performance estimation. Across these repeated experiments, the best performing predictive model identified within the VLCPredictor framework achieved an average AUC of 0.742, indicating that the integrated pipeline effectively captures strain level functional heterogeneity based on genomic k mer features (Table S4).

In the second stage, VLCPredictor was applied to independent external validation datasets lacking Lcris-SFS and Lcris-SubFS labels, with the objective of performing distributional validation of predicted functional potential. For each external application, the predictive model selected during internal evaluation was trained using the full set of 67 labeled strains from the first stage and subsequently used to generate predictions on the external datasets. The first validation cohort consisted of 103 publicly available *L. crispatus* genomes, including 16 isolates from BV patients and 87 from healthy women. In this cohort, strains predicted to have high functional potential were significantly more prevalent in healthy women than in patients with BV, with 5 isolates (31.25%) classified as high functional potential in the disease group and 66 isolates (75.86%) classified as high potential in the healthy group (*P* < 0.05). The second validation cohort comprised 572 *L. crispatus* isolates from this study after excluding the 67 strains used for model development, all of which were derived from healthy women. When the same model trained on the 67 labeled strains was applied to this cohort, 260 isolates (45.45%) were predicted to SFS (Table S5). Collectively, these distributional validation results indicate that *L. crispatus* strains with high predicted functional potential are preferentially associated with healthy vaginal environments, supporting the utility of VLCPredictor as an integrated strain level prediction framework for probiotic candidate prioritization.

## Discussion

In this study, we developed VLCPredictor, a machine-learning framework for rapid prioritization of vaginal *L. crispatus* isolates with superior functional performance. The model extracted genomic k-mer features from 67 *L. crispatus* isolates and integrated five in vitro phenotypic traits (growth capacity, acid production, lactic acid production, H_2_O_2_ production, and antagonistic activity against pathogens), VLCPredictor achieved an AUC of 0.742 for predicting overall functional potential. We further applied the model to an expanded dataset by combining publicly available genomes with those generated in this study, yielding 675 *L. crispatus* genomes (including 103 publicly available and 572 generated in this study) for prediction. The results showed that functionally superior strains were markedly more prevalent in healthy individuals than in BV cohorts. Collectively, VLCPredictor is positioned as an early-stage screening tool that can shorten experimental lead time and reduce the costs of large-scale cultivation and phenotypic screening, thereby facilitating candidate *L. crispatus* selection for downstream validation in the context of women’s reproductive-tract health.

Recent large-scale genomic surveys of lactic acid bacteria (LAB) have compiled a database of 19,699 food-derived strains, revealing broad functional diversity in metabolism and antimicrobial peptide biosynthesis. They also link LAB community features to disease associations and health-enriched markers, underscoring their potential in maintaining microecological balance and supporting disease prevention [53]. Although lactic acid bacteria (LAB) have been extensively studied across diverse niches, most available isolates and genomic resources are derived from food or gastrointestinal contexts, whereas strain-resolved investigations tailored to vaginal health remain comparatively limited. A growing body of evidence suggests that *L. crispatus* plays an important role in modulating vaginal microbial dysbiosis. However, substantial heterogeneity can exist among strains within the same species.

To enrich the vaginal* L. crispatus* resource, we recruited 36 healthy volunteers and obtained 639 *L. crispatus* isolates from 24 of them. Phylogenetic analysis partitioned these isolates into 47 clades, with unique genes concentrated in a minority of clades, suggesting that the species is broadly conserved while still harboring functionally relevant within-species diversity. We further annotated genes encoding antimicrobial peptides and pullulanase in the genomes. Pullulanase can mediate host glycogen degradation, facilitating the growth of *L. crispatus* in the vaginal environment where glycogen serves as a major carbon source, and may promote its dominance and colonization by maintaining low pH and inhibiting pathogens such as *G. vaginalis.* Therefore, selecting strains carrying pulA may be advantageous for vaginal *L. crispatus* development [48, 54]. In recent years, antimicrobial peptides derived from commensal bacteria have been considered important regulators of microecological homeostasis. For example, King et al. screened 2,229 microbial genomes distributed across multiple human body sites and identified more than 200 novel RiPPs, some of which exhibit inhibitory activity against drug-resistant pathogens such as VRE and MRSA and can modulate microbiota composition across different body sites [55]. In the present study, the number and types of pullulanase and RiPP-related genes carried by different strains differed significantly, indicating substantial functional diversity within the species. However, genome annotations alone remain insufficient to accurately capture the actual functional performance of strains, and systematic in vitro experiments are therefore still required for validation.

A *Lactobacillus*-dominated vaginal microbiome is a phenomenon unique to humans, whereas the vaginal microbiota of other primates is predominantly composed of anaerobic bacteria [56]. Acidic conditions are essential for maintaining vaginal health. *L. crispatus* sustains vaginal acidity (pH < 4.2) and inhibits the growth of pathogenic bacteria by catabolizing glycogen and producing lactic acid [56]. H₂O₂ exerts antimicrobial activity by inducing oxidative stress that directly inhibits or kills pathogens, and it can also modulate host immunity by stimulating vaginal epithelial cells to produce antimicrobial peptides (e.g., defensins) and cytokines (e.g., IL-8), thereby regulating NF-κB signaling and limiting excessive pro-inflammatory mediators (e.g., TNF-α and IL-1β) [57]. In addition, H₂O₂ can disrupt biofilms formed by pathogens such as G. vaginalis, supporting vaginal microbial homeostasis [24]. Numerous studies have demonstrated that vaginal LAB exhibit potent antibacterial activity and can inhibit the growth and biofilm formation of pathogenic bacteria, thereby contributing to vaginal microbiota homeostasis [58–60]. Therefore, we selected growth capacity, acid production, lactic acid production, H₂O₂ production and antibacterial ability as key indicators for evaluating strain functional potential. In vitro experiments revealed that although certain strains performed well in individual indicators, a single parameter was insufficient to comprehensively evaluate probiotic potential. We further developed a weighted scoring system that integrates multiple in vitro indicators according to their relative importance. Based on this system, three *L. crispatus* with the highest comprehensive functional potential were identified, which exhibited robust growth and produced sufficient metabolites to effectively inhibit the growth of common vaginal pathogens. These candidate strains are currently being evaluated in ongoing in vivo studies to test whether they can indeed ameliorate BV-related outcomes.

Compared with previous study that focus on cross-species classification or rely on a limited set of known genetic markers and rule-based screening, VLCPredictor is designed to capture within-species variation that is directly tied to phenotypes. Notably, Lcris-SFS defined in this study is not intended to simply label *L. crispatus* isolates as “good” or “bad”. It is an integrative phenotype-based label derived from multiple in vitro readouts relevant to vaginal health maintenance and BV-related functional mechanisms. VLCPredictor is intended as an early-stage screening tool to prioritize, under resource constraints, vaginal *L. crispatus* isolates with relatively higher functional potential from a large collection, thereby reducing the cost and workload of subsequent in vitro screening. Although this workflow is portable and could be extended to other candidate probiotic species, VLCPredictor should not be directly applied to predict other species. Instead, within the research context of the target organism, phenotype and label definitions aligned with the specific research question (e.g., metrics related to particular disease outcomes, niche adaptation, or key functional mechanisms) must be re-established, followed by re-training, calibration, and external validation based on sufficiently large genome and phenotype datasets. Model interpretability and generalizability may be affected when functional labels are ill-defined, cohorts are highly heterogeneous, or markedly distinct disease subtypes are pooled.

In addition, VLCPredictor does not infer functional potential solely from genome annotation. It is trained using in vitro phenotypic measurements and a weighted scoring scheme as supervised signals, thereby learning the correspondence between genomic features and observed functional phenotypes. We further adopt k-mers as the primary input representation, enabling direct extraction of sequence-derived information without fully relying on gene- or pathway-level annotations. Because annotation pipelines, database versions, and parameter settings often vary across studies, features derived from gene/pathway annotations may reduce cross-study comparability. In contrast, k-mer features are derived directly from the genome sequence and can capture strain-level differences even in the presence of structural variation, gene loss/rearrangements, or noncoding variation, which may improve the stability and usability of the model across cohorts and analytical workflows.

Although VLCPredictor has demonstrated promising results in strain-level functional prediction, several limitations persist. Our current external validation was primarily performed using independent isolate genomes available from public databases. We observed that, among these publicly available samples, *L. crispatus* from healthy women were more frequently predicted to have Lcris-SFS, whereas the proportion of such strains was lower among BV-associated isolates. Notably, the Lcris-SFS proportions differ substantially between the public database and our isolate collection. This discrepancy may arise because publicly available *L. crispatus* isolates from healthy women are often influenced by study objectives and curation strategies, which may preferentially enrich for representative strains that are easier to culture, have higher assembly quality, or are more strongly associated with health, thereby yielding a higher predicted Lcris-SFS proportion. Meanwhile, the Lcris-SFS proportion in our isolate collection may also be affected by uneven numbers of isolates recovered from different participants. VLCPredictor operates at the earliest stage of the probiotic development pipeline and currently outputs only a strain-level ranking of functional potential; it cannot directly substitute for assessments of in vivo effects or clinical efficacy. Therefore, candidate strains prioritized by the model still require further validation through animal and clinical studies. During the model validation, the relatively limited availability of whole-genome data volume for *L. crispatus* isolates resulted in a pronounced imbalance between strains from healthy individuals and those from patients with bacterial vaginosis (only 16 BV patients). Furthermore, the validation cohort was restricted to a single geographic and ethnic background, making it difficult to exclude potential biases arising from host genetic, behavioral, and environmental differences. In addition, because we did not include isolates from body sites other than the vagina or from non-human hosts, we do not infer cross-niche generalizability. These factors may limit the generalizability and robustness of the model across diverse populations. Future studies should incorporate genomic data from strains isolated across diverse populations, geographic regions, and vaginal health statuses, and integrate these data with additional in vitro and in vivo functional validations to further enhance the accuracy and applicability of the model. Overall, VLCPredictor provides a standardized workflow for screening vaginal *L. crispatus* isolates and serves as an early-stage predictive model to prioritize candidates for subsequent in vitro and in vivo validation. We anticipate that this framework will not only improve the efficiency of candidate probiotic development in the context of women’s reproductive health, but also offer a transferable methodological blueprint for prioritizing strains associated with other diseases or ecological niches.

## Supplementary Information

Supplementary Material 1.

## Data Availability

The raw sequencing data are publicly available in the China National GeneBank Sequence Archive (CNGBdb; https://db.cngb.org) under accession number CNP0008425. Due to the repository’s controlled-access policy for datasets that may contain human-derived sequences, this dataset is not available for direct public download. Access can be granted upon application through the repository’s controlled-access procedure or by contacting the corresponding/first author.

## Code availability

The source code for the machine learning framework VLCPredictor, including feature extraction, multi-stage feature selection, model training, and prediction modules, is publicly available. The version corresponding to the analyses reported in this study has been archived at Zenodo and can be accessed at 10.5281/zenodo.18149529. The codebase may be further updated and maintained, and the latest version is available on GitHub at https://github.com/LeoWish/VLCPredictor. All scripts required to reproduce the main analyses described in this study are provided in the archived version.

## References

[1] Kennedy MS, Chang EB. The microbiome: composition and locations. Prog Mol Biol Transl Sci. 2020;176:1–42. 33814111 10.1016/bs.pmbts.2020.08.013 PMC8025711

[2] Ayariga JA, Ibrahim I, Gildea L, Abugri J, Villafane R. Microbiota in a long survival discourse with the human host. Arch Microbiol. 2023;205(1):5. 10.1007/s00203-022-03342-6 36441284

[3] Hertzberger R, Morselli S, Botschuijver S, Himschoot L, Steenbergen L, Bruisten S, et al. Degradation of Resistant α-1, 4-glucan by Vaginal Gardnerella Species is Associated with Bacterial Vaginosis. Curr Microbiol. 2025;82(10):1–9. 10.1007/s00284-025-04459-9 PMC12405377 40892095

[4] Rosca AS, Castro J, Sousa LG, Cerca N. *Gardnerella* and vaginal health: the truth is out there. FEMS Microbiol Rev. 2020;44(1):73–105. 31697363 10.1093/femsre/fuz027

[5] Zhu B, Tao Z, Edupuganti L, Serrano MG, Buck GA. Roles of the microbiota of the female reproductive tract in gynecological and reproductive health. Microbiol Mol Biol Rev. 2022;86(4):e00181-21. 36222685 10.1128/mmbr.00181-21 PMC9769908

[6] Gootenberg DB, Mitchell CM, Kwon DS. Cervicovaginal microbiota and reproductive health: the virtue of simplicity. Cell Host Microbe. 2018;23(2):159–68. 29447695 10.1016/j.chom.2018.01.013

[7] Mahajan G, Doherty E, To T, Sutherland A, Grant J, Junaid A, et al. Vaginal microbiome-host interactions modeled in a human vagina-on-a-chip. Microbiome. 2022;10(1):201. 36434666 10.1186/s40168-022-01400-1 PMC9701078

[8] Łaniewski P, Herbst-Kralovetz MM. Bacterial vaginosis and health-associated bacteria modulate the immunometabolic landscape in 3D model of human cervix. NPJ Biofilms Microbiomes. 2021;7(1):88. 34903740 10.1038/s41522-021-00259-8 PMC8669023

[9] Ravel J, Gajer P, Abdo Z, Schneider GM, Koenig SS, McCulle SL, et al. Vaginal microbiome of reproductive-age women. Proceed National Acad Sci. 2011;108(supplement_1):4680–7. 10.1073/pnas.1002611107 PMC3063603 20534435

[10] Lamont RF, Sobel JD, Akins RA, Hassan SS, Chaiworapongsa T, Kusanovic JP, et al. The vaginal microbiome: new information about genital tract flora using molecular based techniques. BJOG. 2011;118(5):533–49. 21251190 10.1111/j.1471-0528.2010.02840.x PMC3055920

[11] Miller EA, Beasley DE, Dunn RR, Archie EA. *Lactobacilli* dominance and vaginal pH: why is the human vaginal microbiome unique? Front Microbiol. 2016;7:1936. 28008325 10.3389/fmicb.2016.01936 PMC5143676

[12] Gajer P, Brotman RM, Bai G, Sakamoto J, Schütte UM, Zhong X, et al. Temporal dynamics of the human vaginal microbiota. Sci Transl Med. 2012;4(132):132ra52-ra52. 10.1126/scitranslmed.3003605 PMC3722878 22553250

[13] France M, Alizadeh M, Brown S, Ma B, Ravel J. Towards a deeper understanding of the vaginal microbiota. Nat Microbiol. 2022;7(3):367–78. 35246662 10.1038/s41564-022-01083-2 PMC8910585

[14] Onderdonk AB, Delaney ML, Fichorova RN. The human microbiome during bacterial vaginosis. Clin Microbiol Rev. 2016;29(2):223–38. 26864580 10.1128/CMR.00075-15 PMC4786887

[15] Van De Wijgert JH. The vaginal microbiome and sexually transmitted infections are interlinked: consequences for treatment and prevention. PLoS Med. 2017;14(12):e1002478. 29281632 10.1371/journal.pmed.1002478 PMC5744905

[16] Eastment MC, McClelland RS. Vaginal microbiota and susceptibility to HIV. AIDS. 2018;32(6):687–98. 29424773 10.1097/QAD.0000000000001768 PMC5957511

[17] Tamarelle J, Thiébaut AC, De Barbeyrac B, Bebear C, Ravel J, Delarocque-Astagneau E. The vaginal microbiota and its association with human papillomavirus, *Chlamydia trachomatis*, *Neisseria gonorrhoeae* and *Mycoplasma genitalium* infections: a systematic review and meta-analysis. Clin Microbiol Infect. 2019;25(1):35–47. 29729331 10.1016/j.cmi.2018.04.019 PMC7362580

[18] Ziklo N, Vidgen ME, Taing K, Huston WM, Timms P. Dysbiosis of the vaginal microbiota and higher vaginal kynurenine/tryptophan ratio reveals an association with *Chlamydia trachomatis* genital infections. Front Cell Infect Microbiol. 2018;8:1. 29404279 10.3389/fcimb.2018.00001 PMC5778109

[19] Feehily C, Crosby D, Walsh CJ, Lawton EM, Higgins S, McAuliffe FM, et al. Shotgun sequencing of the vaginal microbiome reveals both a species and functional potential signature of preterm birth. NPJ biofilms and microbiomes. 2020;6(1):50. 33184260 10.1038/s41522-020-00162-8 PMC7665020

[20] Elovitz MA, Gajer P, Riis V, Brown AG, Humphrys MS, Holm JB, et al. Cervicovaginal microbiota and local immune response modulate the risk of spontaneous preterm delivery. Nat Commun. 2019;10(1):1305. 30899005 10.1038/s41467-019-09285-9 PMC6428888

[21] Grewal K, Lee YS, Smith A, Brosens JJ, Bourne T, Al-Memar M, et al. Chromosomally normal miscarriage is associated with vaginal dysbiosis and local inflammation. BMC Med. 2022;20(1):38. 35090453 10.1186/s12916-021-02227-7 PMC8796436

[22] Zanella R, Camargo Jd, Scariot CA, Marques MG. The microbiome effect on the female reproductive performance. Anim Reprod. 2024;21(3):e20240063. 39175996 10.1590/1984-3143-AR2024-0063 PMC11340800

[23] Hugerth LW, Krog MC, Vomstein K, Du J, Bashir Z, Kaldhusdal V, et al. Defining Vaginal Community Dynamics: daily microbiome transitions, the role of menstruation, bacteriophages, and bacterial genes. Microbiome. 2024;12(1):153. 39160615 10.1186/s40168-024-01870-5 PMC11331738

[24] Han Y, Ren Q-l. Does probiotics work for bacterial vaginosis and vulvovaginal candidiasis. Curr Opin Pharmacol. 2021;61:83–90. 34649216 10.1016/j.coph.2021.09.004

[25] Reznichenko H, Henyk N, Maliuk V, Khyzhnyak T, Tynna Y, Filipiuk I, et al. Oral intake of lactobacilli can be helpful in symptomatic bacterial vaginosis: A randomized clinical study. J Low Genit Tract Dis. 2020;24(3):284–9. 32091443 10.1097/LGT.0000000000000518

[26] van De Wijgert JH, Verwijs MC, Agaba SK, Bronowski C, Mwambarangwe L, Uwineza M, et al. Intermittent lactobacilli-containing vaginal probiotic or metronidazole use to prevent bacterial vaginosis recurrence: a pilot study incorporating microscopy and sequencing. Sci Rep. 2020;10(1):3884. 32127550 10.1038/s41598-020-60671-6 PMC7054572

[27] Webb L. Probiotics for preventing recurrent bacterial vaginosis. Jaapa. 2021;34(2):19–22. 33448711 10.1097/01.JAA.0000731484.81301.58

[28] Sobel JD, Sobel R. Current and emerging pharmacotherapy for recurrent bacterial vaginosis. Expert Opin Pharmacother. 2021;22(12):1593–600. 33750246 10.1080/14656566.2021.1904890

[29] Cohen CR, Wierzbicki MR, French AL, Morris S, Newmann S, Reno H, et al. Randomized trial of lactin-V to prevent recurrence of bacterial vaginosis. N Engl J Med. 2020;382(20):1906–15. 32402161 10.1056/NEJMoa1915254 PMC7362958

[30] Van Der Veer C, Hertzberger RY, Bruisten SM, Tytgat HL, Swanenburg J, de Kat A-B, et al. Comparative genomics of human Lactobacillus crispatus isolates reveals genes for glycosylation and glycogen degradation: implications for in vivo dominance of the vaginal microbiota. Microbiome. 2019;7(1):49. 30925932 10.1186/s40168-019-0667-9 PMC6441167

[31] Zhang Y, Lyu J, Ge L, Huang L, Peng Z, Liang Y, et al. Probiotic *Lacticaseibacillus rhamnosus* GR-1 and *Limosilactobacillus reuteri* RC-14 as an adjunctive treatment for bacterial vaginosis do not increase the cure rate in a Chinese cohort: a prospective, parallel‐group, randomized, controlled study. Front Cell Infect Microbiol. 2021;11:669901. 34295831 10.3389/fcimb.2021.669901 PMC8291149

[32] Lebeer S, Ahannach S, Gehrmann T, Wittouck S, Eilers T, Oerlemans E, et al. A citizen-science-enabled catalogue of the vaginal microbiome and associated factors. Nat Microbiol. 2023;8(11):2183–95. 37884815 10.1038/s41564-023-01500-0 PMC10627828

[33] Bloom S, Mafunda N, Woolston B, Hayward M, Frempong J, Abai A, et al. Cysteine dependence of Lactobacillus iners is a potential therapeutic target for vaginal microbiota modulation. Nat Microbiol. 2022;7:434–50. 10.1038/s41564-022-01070-7. 35241796 10.1038/s41564-022-01070-7 PMC10473153

[34] Petrova MI, Reid G, Vaneechoutte M, Lebeer S. *Lactobacillus iners*: friend or foe? Trends Microbiol. 2017;25(3):182–91. 27914761 10.1016/j.tim.2016.11.007

[35] Fontana F, Alessandri G, Lugli GA, Mancabelli L, Longhi G, Anzalone R, et al. Probiogenomics analysis of 97 *Lactobacillus crispatus* strains as a tool for the identification of promising next-generation probiotics. Microorganisms. 2020;9(1):73. 33396617 10.3390/microorganisms9010073 PMC7824148

[36] Jenkins DJ, Woolston BM, Hood-Pishchany MI, Pelayo P, Konopaski AN, Quinn Peters M, et al. Bacterial amylases enable glycogen degradation by the vaginal microbiome. Nat Microbiol. 2023;8(9):1641–52. 37563289 10.1038/s41564-023-01447-2 PMC10465358

[37] Han X, Liu Q, Li Y, et al. Synergizing artificial intelligence and probiotics: a comprehensive review of emerging applications in health promotion and industrial innovation. Trends Food Sci Technol. 2025;159:104938.

[38] Sun Y, Li H, Zheng L, Li J, Hong Y, Liang P, et al. iProbiotics: a machine learning platform for rapid identification of probiotic properties from whole-genome primary sequences. Brief Bioinform. 2022;23(1):bbab477. 34849572 10.1093/bib/bbab477

[39] Wu S, Feng T, Tang W, Qi C, Gao J, He X, et al. metaProbiotics: a tool for mining probiotic from metagenomic binning data based on a language model. Brief Bioinform. 2024;25(2):085. 10.1093/bib/bbae085 PMC10940841 38487846

[40] Javdan B, Lopez JG, Chankhamjon P, Lee YCJ, Hull R, Wu Q, et al. Personalized mapping of drug metabolism by the human gut microbiome. Cell. 2020;181(7):1661-79. e22. 32526207 10.1016/j.cell.2020.05.001 PMC8591631

[41] Bankevich A, Nurk S, Antipov D, Gurevich AA, Dvorkin M, Kulikov AS, et al. SPAdes: a new genome assembly algorithm and its applications to single-cell sequencing. J Comput Biol. 2012;19(5):455–77. 22506599 10.1089/cmb.2012.0021 PMC3342519

[42] Parks DH, Imelfort M, Skennerton CT, Hugenholtz P, Tyson GW. CheckM: assessing the quality of microbial genomes recovered from isolates, single cells, and metagenomes. Genome Res. 2015;25(7):1043–55. 25977477 10.1101/gr.186072.114 PMC4484387

[43] Jain C, Rodriguez-R LM, Phillippy AM, Konstantinidis KT, Aluru S. High throughput ANI analysis of 90K prokaryotic genomes reveals clear species boundaries. Nat Commun. 2018;9(1):5114. 30504855 10.1038/s41467-018-07641-9 PMC6269478

[44] Seemann T. Prokka: rapid prokaryotic genome annotation. Bioinformatics. 2014;30(14):2068–9. 24642063 10.1093/bioinformatics/btu153

[45] Zheng J, Ge Q, Yan Y, Zhang X, Huang L, Yin Y. dbCAN3: automated carbohydrate-active enzyme and substrate annotation. Nucleic Acids Res. 2023;51(W1):W115–21. 37125649 10.1093/nar/gkad328 PMC10320055

[46] Alcock BP, Huynh W, Chalil R, Smith KW, Raphenya AR, Wlodarski MA, et al. CARD 2023: expanded curation, support for machine learning, and resistome prediction at the Comprehensive Antibiotic Resistance Database. Nucleic Acids Res. 2023;51(D1):D690–9. 36263822 10.1093/nar/gkac920 PMC9825576

[47] Blin K, Shaw S, Augustijn HE, Reitz ZL, Biermann F, Alanjary M, et al. antiSMASH 7.0: new and improved predictions for detection, regulation, chemical structures and visualisation. Nucl Acids Res. 2023;51(W1):W46–50. 37140036 10.1093/nar/gkad344 PMC10320115

[48] Jenkins D, Woolston B, Hood-Pishchany M, Pelayo P, Konopaski A, Quinn Peters M, et al. Bacterial amylases enable glycogen degradation by the vaginal microbiome. Nat Microbiol. 2023;8:1641–52. 37563289 10.1038/s41564-023-01447-2 PMC10465358

[49] UniProt. the universal protein knowledgebase in 2023. Nucl Acids Res. 2023;51(D1):D523–31. 36408920 10.1093/nar/gkac1052 PMC9825514

[50] Hyatt D, Chen G-L, LoCascio PF, Land ML, Larimer FW, Hauser LJ. Prodigal: prokaryotic gene recognition and translation initiation site identification. BMC Bioinformatics. 2010;11(1):119. 20211023 10.1186/1471-2105-11-119 PMC2848648

[51] Asnicar F, Thomas AM, Beghini F, Mengoni C, Manara S, Manghi P, et al. Precise phylogenetic analysis of microbial isolates and genomes from metagenomes using PhyloPhlAn 3.0. Nat Commun. 2020;11(1):2500. 32427907 10.1038/s41467-020-16366-7 PMC7237447

[52] Stamatakis A. RAxML version 8: a tool for phylogenetic analysis and post-analysis of large phylogenies. Bioinformatics. 2014;30(9):1312–3. 24451623 10.1093/bioinformatics/btu033 PMC3998144

[53] Jin H, Quan K, You L, Kwok L-Y, Ma T, Li Y, et al. A genomic compendium of cultivated food-derived lactic acid bacteria unveils their contributions to human health. Sci Bull. 2025;70(11):1761–5. 10.1016/j.scib.2024.12.002 39672713

[54] Jenkins DJ, Woolston BM, Hood-Pishchany MI, Pelayo P, Konopaski AN, Peters MQ, et al. Identification and characterization of bacterial glycogen-degrading enzymes in the vaginal microbiome. bioRxiv. 2021:2021.07. 19.452977.

[55] King AM, Zhang Z, Glassey E, Siuti P, Clardy J, Voigt CA. Systematic mining of the human microbiome identifies antimicrobial peptides with diverse activity spectra. Nat Microbiol. 2023;8(12):2420–34. 37973865 10.1038/s41564-023-01524-6

[56] Witkin SS, Linhares IM. Why do lactobacilli dominate the human vaginal microbiota? BJOG. 2017;124(4):606–11. 28224747 10.1111/1471-0528.14390

[57] Miko E, Barakonyi A. The role of hydrogen-peroxide (H_2_O_2_) produced by vaginal microbiota in female reproductive health. Antioxidants. 2023;12(5):1055. 37237921 10.3390/antiox12051055 PMC10215881

[58] Liu P, Lu Y, Li R, Chen X. Use of probiotic lactobacilli in the treatment of vaginal infections: in vitro and in vivo investigations. Front Cell Infect Microbiol. 2023;13:1153894. 37077531 10.3389/fcimb.2023.1153894 PMC10106725

[59] Chee WJY, Chew SY, Than LTL. Vaginal microbiota and the potential of *Lactobacillus* derivatives in maintaining vaginal health. Microb Cell Fact. 2020;19(1):203. 33160356 10.1186/s12934-020-01464-4 PMC7648308

[60] Huang J, Zheng L, Su Y, Wang F, Kong H, Chang Y, et al. Effects of group B streptococcus infection on vaginal micro-ecology and pregnancy outcomes of pregnant women in late pregnancy. Eur J Obstet Gynecol Reprod Biol. 2021;267:274–9. 34839249 10.1016/j.ejogrb.2021.11.419

